# A caffeine and theacrine combination improves cognitive performance in tactical personnel under physically fatiguing conditions

**DOI:** 10.1080/15502783.2025.2536146

**Published:** 2025-07-22

**Authors:** Blaine S. Lints, Adam T. Harrison, Sten O. Stray-Gundersen, Gianna F. Mastrofini, Riccardo F. Romersi, Noah K. Nakagawa, Mackenzie B. Yoder, Chimaobim E. Martin-Diala, Alexa J. Chandler, R. Davis Moore, Shawn M. Arent

**Affiliations:** a Department of Exercise Science, Arnold School of Public Health, University of South Carolina, Columbia, SC, USA; b Department of Movement Sciences, College of Education, Health and Human Sciences, University of Idaho, Moscow, ID, USA

**Keywords:** Sports nutrition, dietary supplements, stimulants, reaction time

## Abstract

**Background:**

Optimizing human performance under stressful physical and cognitive conditions is paramount during high-stakes military operations. As such, interventions to improve warfighter performance by mitigating task-induced cognitive performance deficits are necessary. While caffeine is the most widely consumed psychoactive substance in the world, common side effects (i.e. anxiousness, micro-saccades, irritability) may be detrimental to warfighter operations. Theacrine, a purine alkaloid similar in structure to caffeine but with a longer half-life, less habituation, and fewer side effects, is proposed as a caffeine-alternative to enhance cognitive resilience.

**Methods:**

Tactically trained participants (*n* = 20; F = 5; *M* = 16; age = 21.5 ± 3.8 y) completed one baseline and three experimental visits. Baseline included familiarization with cognitive tasks and a graded exercise test to determine VO_2max_. Experimental sessions, separated by ≥96 hours, were double-blind, placebo-controlled, and randomized. Participants completed cognitive testing (Dynavision, Trazer, Object Hit and Avoid, Anti-Saccade, Two-Back), consumed either 300 mg caffeine (CAF), 150 mg caffeine +150 mg theacrine (CTC), or placebo (PLA), and repeated cognitive tests 60-min post supplementation. After a high-intensity interval exercise session (10 intervals at >90% VO_2max_ with recovery), reaction time (RT) tests were repeated after the 4th and 8th intervals, as well as immediately and 30-min post-exercise. Heart rate was measured continuously. Change scores were calculated from baseline, and data analyzed using repeated measures ANOVA (α = 0.05) with Bonferroni correction.

**Results:**

Both CAF and CTC consistently enhanced cognitive and physical performance compared to PLA. In the Two-Back task, CTC showed significantly higher total accuracy (*p* < 0.01), while both CAF and CTC had fewer target (*p* < 0.01) and non-target errors (CAF: *p* = 0.03; CTC: *p* < 0.01) than PLA. CTC also had significantly faster RTs (*p* = 0.03) and lower RT variability for non-target trials compared to both PLA and CAF (*p* < 0.01). For the Dynavision Go/NoGo task, both CTC (*p* = 0.01) and CAF (*p* = 0.03) had significantly faster RTs than PLA, regardless of time point. Post-exercise, accuracy (*p* = 0.01) and RTs (*p* < 0.01) improved significantly compared to post-supplement. In the Object Hit & Avoid task, CAF and CTC significantly improved task accuracy (*p* < 0.01) while reducing omission (*p* < 0.01) and commission errors (*p* < 0.01) compared to PLA. HRV measures (RMSSD, NN intervals, SDNN) significantly increased post-supplementation (*p* < 0.01) but decreased immediately (*p* < 0.01) and 30 min post-exercise (*p* < 0.01). Blood lactate significantly declined at 5 (*p* < 0.01) and 10 min (*p* < 0.01) post-exercise compared to immediately post-exercise.

**Conclusion:**

SIngestion of CAF and CTC improved various measures of cognitive performance before, and after fatiguing exercise. Furthermore, CTC had additional cognitive benefits beyond CAF. Thus, combining lower-dose caffeine and theacrine may improve cognitive-behavioral performance before and after fatiguing exercise to an equal or greater degree than higher doses of caffeine alone. This combination offers a non-pharmacological intervention for those who experience side effects with caffeine to mitigate the impact of physical and cognitive stress. Future research should examine higher doses of theacrine alone or with caffeine, chronic supplementation, extended exercise durations, and different cognitive metrics under varied stress and environmental conditions.

## 1. Introduction

In the high-stakes environment of tactical operations, optimizing human performance, particularly under conditions of excessive physical and cognitive stress, is paramount. Considering cognitive performance tends to decline in these situations, efficacious interventions to improve warfighter performance are needed [1,2]. As such, dietary supplementation may serve as a promising avenue to bolster cognitive and physical capabilities during high-stakes tactical operations [2].

Caffeine, the world’s most widely consumed psychoactive substance, is well-known for enhancing vigilance, attention, physical endurance, and mood through adenosine inhibition [3–5]. Its ergogenic effects are also well-documented, making it a staple in the repertoire of ergogenic supplements [3]. However, caffeine, particularly in high doses, may be accompanied by various side effects such as undesirable cardiovascular responses, habituation, and anxiety that may negatively impact performance [7,8].

Theacrine, a purine alkaloid similar in structure to caffeine has gained attention for its stimulatory effects that appear to mimic those of caffeine, albeit with a longer half-life and lower incidence of habituation [5]. While theacrine appears to deliver a smoother, more sustained stimulatory effect with fewer negative reactions, it has failed to demonstrate improvements in physical performance when used in isolation [,^6^]. Thus, using a combination of caffeine and theacrine (CTC) may optimize performance enhancement strategies [7].

Recent findings demonstrate that acute CTC supplementation reduces fatigue and improves cognitive performance among high-level soccer players during a simulated match [7]. Similarly, Cintineo and colleagues observed that CTC and caffeine supplementation improved vigilance in tactically trained personnel compared to placebo [8]. Importantly, this study also demonstrated that CTC supplementation did not elicit significant hemodynamic alterations as seen with caffeine supplementation. However, the benefits of CTC before and after physically fatiguing exercise in tactical populations has yet to be explored.

Therefore, the present study sought to determine whether CTC offers a superior alternative to caffeine used in isolation to enhance cognitive performance and sustain performance following a fatiguing exercise protocol. The significance of this study extends beyond the military, offering insights into cognitive and physical performance enhancement strategies that could benefit athletes, first responders, and others in occupations in which critical and timely decisions are required under psychologically and physiologically stressful conditions.

## 2. Methods

A randomized, double-blind, placebo-controlled, within-subjects cross-over design was used to compare the effects of a combination of caffeine (150 mg) + theacrine (150 mg) supplement (CTC), caffeine (300 mg) alone (CAF), and cellulose (300 mg) placebo (PLA) on heart rate, autonomic responses, cognition, and functional performance before and after fatiguing exercise. Study products were provided by Compound Solutions, Inc. (Carlsbad, CA, USA) and third-party tested for verification of identity, purity, and potency (Dyad Labs, Salt Lake City, UT, USA). Average relative caffeine dose was 3.8 ± 0.6 mg/kg for the CAF condition (3.6 ± 0.4 for men and 4.6 ± 0.3 women). The average relative dose of both caffeine and theacrine was 1.9 ± 0.3 mg/kg for the CTC condition (1.8 ± 0.2 for men and 2.3 ± 0.1 for women). Participation in the clinical trial required each participant to complete one familiarization visit and three experimental visits separated by ≥96 hours. For the experimental visits, the supplementation order was randomized for each participant using a random sequence generator in MATLAB 2023b (MathWorks, Natick, MA, USA). All protocols and procedures were approved by the University of South Carolina Institutional Review Board (IRB Number: Pro00123869; approved 4/10/2023). This study was prospectively registered on ClinicalTrials.gov (NCT05715073).

### 2.1. Participants

**Figure 1.**
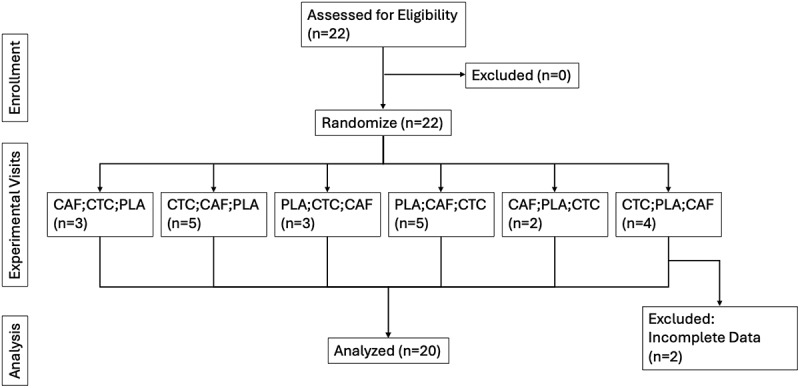
CONSORT diagram.

Twenty current Reserve Officers’ Training Corp (ROTC) cadets or midshipmen, or current military/law enforcement officers completed this clinical trial (*n* = 20; F = 5; *M* = 16; age = 21.5 ± 3.8 y; height = 177.1 ± 8.8 cm; weight = 78.5 ± 15.3 kg; VO_2max_ = 49.6 ± 8.5 ml/kg/min). All participants regularly participated in resistance and/or endurance training (≥4 days/week and ≥150 min/week) for at least the last six months. Participants were excluded if they 1) were currently taking any stimulant medication, 2) had any known metabolic disorder (e.g. electrolyte abnormalities, diabetes, thyroid disease), 3) had a history of hepatorenal, musculoskeletal, autoimmune, or neurological disease, 4) experienced migraines, or 5) had a known caffeine sensitivity or allergy to any ingredient in the supplement or placebo. Each participant was questioned about their dietary supplement use within the last six months. Any participant beginning a new supplement routine within the last month was asked to discontinue use and allow for a two-week washout before participation. In all other cases, participants were asked to maintain their typical supplement routine throughout the study. All subjects provided written informed consent, and the study was approved by the University of South Carolina Institutional Review Board. A CONSORT diagram is presented in Figure 1.

### 2.2. Familiarization procedure

During familiarization, participants completed questionnaires on caffeine intake, physical activity, and health to verify inclusion. They were briefed on the study protocols, provided informed consent, and familiarized with cognitive and physical testing (Dynavision Go/NoGo, Trazer Flanker, and KINARM assessments). A graded treadmill test was used to determine VO_2max_ and set exercise intensities for the experimental visits. To standardize visit conditions, participants were asked to refrain from eating for two hours prior, and from caffeine or alcohol ingestion, and vigorous exercise for 24 hours prior. Adherence to these restrictions was confirmed verbally for each visit. While the exact composition and timing of the last meal before each experimental visit were not standardized, participants were encouraged to replicate their dietary intake before each session and compliance was verbally confirmed. This approach was used to balance experimental control with participant feasibility while minimizing acute postprandial influences.

### 2.3. Experimental testing procedure

**Figure 2.**
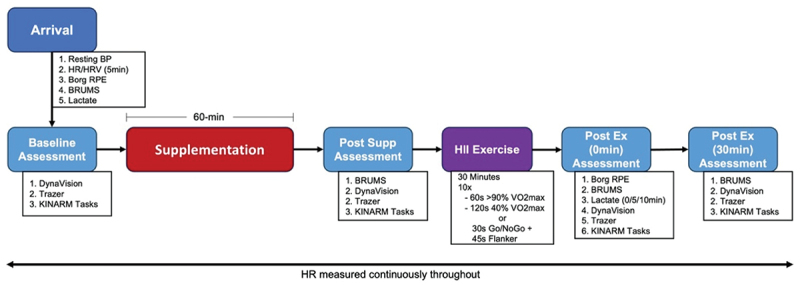
Flow chart of timeline of measurements.

Figure 2 provides a visual outline of the experimental visit procedure. Participants were asked to sit comfortably and quietly in a chair prior to baseline blood pressure and heart rate recordings. A five-min resting heart rate (HR) recording was obtained via a Polar chest strap (Polar Electro, Kempele, Finland). HR was continuously recorded throughout the remainder of the experimental session. Following the resting HR assessment, participants were asked to rate their perceived exertion on the Borg RPE scale and provided a baseline blood lactate sample [9]. Capillary blood samples (5 mL) were taken from the fingertip to analyze blood lactate concentrations. The Lactate Plus (Nova Biomedical, Waltham, MA, USA) portable analyzer was used to determine whole-blood lactate content.

Participants then underwent a baseline comprehensive cognitive assessment battery identical to the tasks completed during the familiarization visit. Following the battery of cognitive assessments, participants consumed their randomized supplement (CAF/CTC/PLA). Participants then completed a 60 min waiting period in which they were instructed to sit and leisurely read. At the 45 min mark of the 60 min waiting period, participants repeated the battery of cognitive assessments. After finishing the post supplement assessment, participants performed a 30 min high-intensity interval (HII) exercise protocol. The HII exercise protocol consisted of ten repetitions of 60s high-intensity intervals (≥90% VO_2max_) followed by 120s low-intensity intervals (~40% VO_2max_). To mimic the fluctuating cognitive and physical demands necessary for tactical athletes, the 4th and 8th low-intensity interval was replaced with functional cognitive tasks (Dynavision Go/NoGo and Trazer Flanker task). Participants then repeated the cognitive assessment battery immediately following exercise and 30 min post-exercise. Additionally, blood lactate samples were collected immediately following exercise, 5 min, and 10 min post-exercise. All visits were separated by a minimum of 96 hours.

A typical experimental visit lasted approximately two hours. Based on the testing schedule, cognitive assessments occurred approximately 60 min, 95 min, and 125 min following supplement ingestion.

### 2.4. Cognitive assessment battery

During each cognitive assessment, participants completed the Dynavision Go/NoGo task, Trazer Flanker task, and a series of tasks of executive function tasks within the KINARM End-Point system. In total, the cognitive assessment battery took approximately 15 min to complete.

#### 2.4.1. Dynavision – Go/NoGo task

Participants completed a Go/NoGo reaction time test on a Dynavision D2™ Reaction Board (D2, Dynavision International LLC, West Chester, OH, USA). The Dynavision D2™ is an interactive light board with 64 three-dimensional targets. Participants were instructed to hit the red light as the “Go” stimulus targets (80%) as fast as possible. Green targets were indicative of a “NoGo” stimulus (20%) and were to be avoided. The Dynavision D2™ recorded the participants’ average reaction time to all the red targets and the number of green targets hit. Total task time was approximately 30 seconds. Total accuracy (%) and Go-target reaction time (RT; ms) were recorded for each task run. Average baseline coefficient of variation (CV) was 4% and 5% for accuracy and RT, respectively.

#### 2.4.2. TRAZER – Eriksen Flanker task

Participants completed a full body variant of the Eriksen Flanker task within the Trazer motion capture system (Trazer, TRAQ Global Ltd., Westlake, OH, USA). The Trazer utilizes motion capture cameras and projects the participant as a three-dimensional avatar on a screen in front of them. In this augmented reality environment, participants completed a modified version of the Eriksen Flanker task [10] in which they were instructed to make lateral jumping responses in response to a center arrow presented above their avatar on the screen. In addition to the center arrow, flanking non-target arrows were presented. These arrows were either congruent (<<<< < or >>>>>) or incongruent (<<>< < or >><>>) to the central arrow. Participants were given 45 seconds to reach as many targets as quickly and accurately as possible. Average reaction time (CV = 10%) and number of targets reached (CV = 10%) were recorded for each task run.

#### 2.4.3. KINARM

The KINARM End-Point Lab system (BKIN Technologies, Kingston, Canada) with an integrated EyeLink 1000SR remote eye-tracker (SR Research, Ottawa, Canada) was used to assess sensorimotor and cognitive brain function. This setup provides precise, simultaneous measurements of upper limb kinematics and eye movements in a horizontal augmented reality environment. Participants were seated in a custom chair, calibrated individually for accurate eye tracking. A gaze calibration procedure was performed before each session, where participants sequentially looked at visual targets, allowing accurate quantification of gaze behavior during task performance.

##### 2.4.3.1. Object hit and avoid

The Object Hit and Avoid (OHA) task assessed rapid response execution and inhibition [11]. Participants used virtual paddles to hit target objects (*n* = 200) while avoiding distractors (*n* = 100) as they moved toward them. Task difficulty increased with the number and speed of falling objects. Key measures recorded included total accuracy (%) (CV = 4%), omission errors (targets missed, %) (CV = 37%), commission errors (distractors hit, %) (CV = 47%), and total objects foveated (%) (CV = 3%).

##### 2.4.3.2. Anti-/pro-saccade task

The anti-/pro-saccade (APS) task assessed response inhibition, working memory, and visuospatial attention. [12,13] Participants made eye movements toward (pro-saccade) or away (anti-saccade) from stimuli, with conditions indicated by the color of a central fixation stimulus. Pro- and anti-saccade trials were randomly interleaved. Key measures recorded were total accuracy (%) (CV = 7%), pro-/anti-trial errors (%) (pro-trial error CV = 89%; anti-trial error CV = 49%), pro-/anti-trial reaction time (RT, ms) (pro-trial RT CV = 10%; anti-trial RT CV = 10%), and the coefficient of variation of (pro target CVRT CV = 27%; anti target CVRT CV = 22%), with lower CVRT indicating more consistent responses.

##### 2.4.3.3. 2-back task

The 2-back task was used to evaluate working memory capacity [14,15]. Participants responded using a handheld two-button response box, pressing left if the current shape matched the one from two trials ago (target) or right if it was different (non-target). Key measures recorded included total accuracy (%) (CV = 11%), target/non-target errors (%) (target error CV = 26%; non-target error CV = 62%), target/non-target RT (target RT CV = 14%; non-target RT CV = 12%), and target/non-target CVRT (%) (target CVRT CV = 32%; non-target CVRT CV = 16%).

### 2.5. Cardioautonomic function: heart rate variability (HRV)

Continuous HR was recorded using a Polar H10 chest strap (Polar Electro, Finland) at 1000 Hz. Timestamps were manually added via the Polar Team app to mark the start and end of each task. HRV was analyzed for baseline rest, baseline cognitive assessment, post-supplement cognitive assessment, immediately post-exercise, and 30-min post-exercise assessments. HR data were processed in MATLAB 2023b (MathWorks, Natick, MA, USA), calculating RR intervals, applying a 10% hamming window, and removing ectopic beats using a cubic spline algorithm to produce normalized normalized-to-normalized intervals (NN). Time-domain HRV metrics included HRmean (CV = 8%), NNmean (CV = 9%), SDNN (CV = 23%), RMSSD (CV = 30%), and CVNN (CV = 20%), computed over three 5-min segments to ensure consistency across cognitive assessments [16].

### 2.6. Statistical analysis

To account for individual differences, percentage change scores (%Δ) were calculated by subtracting baseline values from subsequent assessments. Descriptive statistics (means ± SD) were computed for %Δ scores. Additionally, effect sizes were calculated as Cohen’s *d*. To evaluate supplementation effects on cognitive performance, HRV, and lactate, condition (PLA, CAF, CTC) by time repeated measures ANOVAs were performed for each respective outcome. Greenhouse-Geisser corrections were applied where sphericity was violated, and Bonferroni-corrected post-hoc analyses were used for significant interactions or main effects. Statistical analyses were conducted using MATLAB 2023b (MathWorks, Natick, MA, USA).

## 3. Results

### 3.1. Dynavision: Go/NoGo

Table 1 shows condition Dynavision task performance at each evaluation time point. No significant condition-by-time effects were observed. A main effect of condition was observed for Dynavision RT (*p* = 0.01). Post-hoc analysis revealed that the CTC condition demonstrated significantly faster RT compared to PLA, irrespective of time point (−3.0%, *p* = 0.01, *d* = −0.52), representing a moderate effect. Similarly, the CAF condition showed significantly faster RT compared to PLA, irrespective of time point (−2.6%, *p* = 0.03, *d* = −0.46), indicating a moderate effect. There were no significant differences in RT between the CAF and CTC conditions. A main effect of time was observed for Dynavision Accuracy (*p* = 0.01) and Dynavision RT (*p* = 0.01). Post-hoc analysis revealed that participants exhibited significantly greater accuracy during the immediate post-exercise cognitive evaluation compared to the post-supplement cognitive evaluation (+3.0%, *p* = 0.01, *d* = 0.66), indicating a moderate effect. However, no significant differences were observed between the post-supplement and 30-min post-exercise evaluations or between the immediate and 30-min post-exercise evaluations. Additionally, participants demonstrated significantly faster RT during the immediate post-exercise cognitive evaluation compared to the post-supplement evaluation (−3.0%, *p* < 0.01, *d* = −0.61), reflecting a moderate effect. No significant differences were found between the post-supplement and 30-min post-exercise evaluations or between the immediate and 30-min post-exercise evaluations.

**Table 1.** Descriptive characteristics at each timepoint for Dynavision accuracy and reaction time (RT, ms). Percent changes were calculated as an average of individual percentage changes at each time point relative to baseline values. For condition effects, A = CAF, B = CTC, and C = PLA. For time effects, numbers represent time points in sequential order.

	CAF					CTC					PLA					Condition		Time		Interaction	
	Mean	SD	*d*	%Δ Mean	%Δ SD	Mean	SD	*d*	%Δ Mean	%Δ SD	Mean	SD	*d*	%Δ Mean	%Δ SD	*p*	η^2^	*p*	η^2^	*p*	η^2^
**Accuracy**																0.341	0.013	**0.007**	**0.057**	0.878	0.007
1. Baseline	95.3	3.8	–	–	–	95.9	2.4	–	–	–	93.5	7.4	–	–	–						
2. Post-Supp	94.9	4.1	–	−1.7	2.6	93.8	4.9	–	−1.8	4.4	92.5	5.1	–	−1.3	6.3			** < 3 & = 4**			
3. Post-Ex	96.1	2.9	−0.78	0.9	4.4	97.8	2.4	−0.89	1.4	3.6	95.7	3.3	−0.32	2.0	5.4			** > 2 & = 4**			
4. 30-m Post-Ex	96.1	3.5	−0.49	0.1	3.9	95.8	4.3	−0.31	−0.7	5.0	94.8	4.5	−0.27	2.1	9.3			** = 2 & = 3**			
**RT (s)**																**0.005**	**0.06**	**0.007**	**0.057**	0.534	0.018
1. Baseline	0.64	0.05	–	–	–	0.65	0.06	–	–	–	0.65	0.07	–	–	–	A = B & A < C					
2. Post-Supp	0.64	0.05	–	0.4	3.9	0.64	0.06	–	−1.6	4.6	0.67	0.08	–	2.6	4.4	B = A & B < C		> 3 & = 4			
3. Post-Ex	0.62	0.05	0.74	−2.8	5.2	0.62	0.05	0.35	−3.6	5.0	0.63	0.05	0.62	−1.6	6.4			< 2 & = 3			
4. 30-m Post-Ex	0.63	0.06	0.50	−2.4	4.8	0.64	0.05	−0.15	−0.9	6.2	0.65	0.06	0.11	1.9	7.0			= 2 & = 3			

### 3.2. Trazer: Flanker task

Table 2 shows condition performance changes from baseline on Trazer task performance at each evaluation time point. No significant condition-by-time effects were present. A main effect of condition was observed for the number of targets reached (*p* < 0.01). Post-hoc analysis showed that both CAF (+4.6%, *p* = 0.02, d = 0.50), representing a moderate effect, and CTC (+5.4%, *p* < 0.01, d = 0.60), also reflecting a moderate effect, acquired significantly more targets compared to PLA, with no significant differences between CAF and CTC conditions (−0.8%, *p* = 0.99, d = −0.05). No other main effects for condition were observed (p’s > 0.05). In addition, no main effects for time were observed (p’s > 0.05).

**Table 2.** Descriptive characteristics at each timepoint for Trazer number of targets reached and reaction time (RT, ms). Percent changes were calculated as an average of individual percentage changes at each time point relative to baseline values. For condition effects, A = CAF, B = CTC, and C = PLA.

	CAF					CTC					PLA					Condition		Time		Interaction	
	Mean	SD	*d*	%Δ Mean	%Δ SD	Mean	SD	*d*	%Δ Mean	%Δ SD	Mean	SD	*d*	%Δ Mean	%Δ SD	p	η^2^	p	η^2^	p	η^2^
**Accuracy**																**0.002**	**0.069**	0.345	0.012	0.845	0.008
1. Baseline	11.6	1.5	-	-	-	11.2	1.4	-	-	-	11.8	1.2	-	-	-	A = B & A > C					
2. Post-Supp	11.8	1.6	-	4.5	8.2	11.7	1.3	-	3.9	6.0	11.6	1.3	-	−1.9	7.9	B = A & B > C					
3. Post-Ex	12.0	1.7	−0.24	5.1	8.7	11.9	1.3	−0.18	6.0	10.4	11.9	1.6	−0.55	2.6	9.2	C < A & C < B					
4. 30-m Post-Ex	12.0	1.3	−0.05	4.1	10.1	11.8	1.2	−0.30	6.2	8.6	11.5	1.4	−0.04	−0.7	10.6						
**RT (s)**																0.665	0.005	0.244	0.163	0.625	0.015
1. Baseline	0.65	0.08	-	-	-	0.67	0.07	-	-	-	0.69	0.08	-	-	-						
2. Post-Supp	0.65	0.09	-	2.3	12.1	0.68	0.07	-	0.9	8.0	0.70	0.07	-	2.9	9.2						
3. Post-Ex	0.64	0.05	0.26	−0.4	11.1	0.66	0.06	0	0.8	11.8	0.67	0.05	0.93	−3.9	6.8						
4. 30-m Post-Ex	0.68	0.09	0.06	2.7	12.8	0.66	0.06	0.05	−0.4	10.2	0.69	0.06	0.18	0.5	12.0						

### 3.3. Object hit & avoid performance

**Figure 3.**
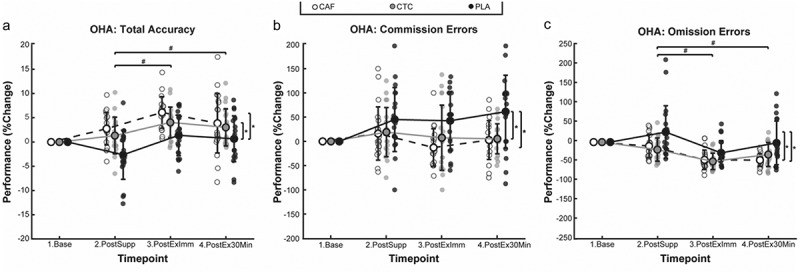
Condition means ± SE at each timepoint for the Object hit and avoid (OHA) task (#*p* < 0.05 by timepoint and *p < 0.05 by condition).

Figure 3 shows condition performance changes from baseline on Object Hit & Avoid performance measures at each evaluation time point. No significant condition-by-time effects were observed.

Main effects of condition were observed for total accuracy (*p* < 0.01), %omission errors (*p* < 0.01), %commission errors (*p* < 0.01), %objects foveated (*p* = 0.01). Post-hoc analysis revealed that both CAF (+4.4%, *p* < 0.01, d = 0.99) and CTC (+3.0%, *p* < 0.01, d = 0.68) conditions demonstrated significantly better task accuracy compared to PLA, indicating moderate to large effects, irrespective of time point, with no significant difference between CAF and CTC. Both CAF and CTC conditions committed significantly fewer omission errors (CAF: −32.8%, *p* < 0.01, d = −0.72 and CTC: −32.4%, *p* < 0.01, d = −0.71) and commission errors (CAF: −48.3%, *p* < 0.01, d = −0.87 and CTC: −39.0%, *p* < 0.01, d = −0.65), representing moderate to large effects and no significant differences between CAF and CTC for omission errors or commission errors. Compared to PLA, CAF also demonstrated a significantly greater percentage of objects foveated (+1.6%, *p* < 0.01, d = 0.58), reflecting moderate effects, though no significant differences were found between CAF and CTC or between CTC and PLA. Main effects of time were observed for total accuracy (*p* < 0.01) and percentage of omission errors (*p* < 0.01). Post-hoc analysis showed significantly increased performance accuracy compared to post-supplement with moderate effects at both immediate post-exercise (+3.4%, *p* < 0.01, d = 0.77) and 30 min post-exercise (+2.0%, *p* = 0.04, d = 0.44), with no significant difference between the two time points. Similarly, participants committed significantly fewer omission errors immediately post-exercise (−39.9%, *p* < 0.01, d = −0.10) and 30 min post-exercise (−25.7%, *p* < 0.01, d = −0.56), indicating small and moderate effects, respectively, compared to post-supplement, with no significant difference between immediate and 30 min post-exercise evaluations.

### 3.4. Anti-/Pro-saccade task performance

**Figure 4.**
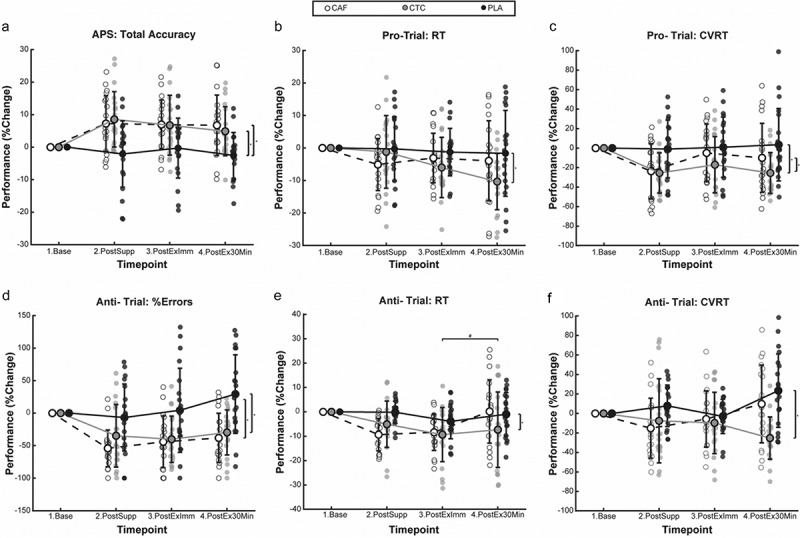
Condition means ± SE at each timepoint for the anti-saccade task (APS) (#*p* < 0.05 by timepoint and *p < 0.05 by condition).

Figure 4 shows condition performance changes from baseline on anti-/pro-saccade performance measures at each evaluation time point. Repeated measures ANOVA revealed a significant condition-by-time interaction for CVRT to anti-saccade targets (*p* < 0.01). Bonferroni-corrected analyses showed no significant differences among conditions at post-supplement or immediate post-exercise (p’s > 0.05). However, at 30-min post-exercise, CVRT was significantly lower in the CTC condition compared to CAF (−35.0%, *p* < 0.01, d = −0.37) and PLA (−48.6%, *p* < 0.01, d = −0.52), exhibiting small to moderate effects. No other interactions were observed for anti-/pro-saccade measures. Main effects of condition were observed for total accuracy (*p* < 0.01), pro-target RT (*p* = 0.03), pro-target CVRT (*p* < 0.01), % anti-target errors (*p* < 0.01), and anti-target RT (*p* < 0.01). Post hoc analysis revealed that compared to PLA, both CAF (+8.6%, *p* < 0.01, d = 1.00) and CTC (+8.4%, *p* < 0.01, d = 0.97) showed significantly greater total accuracy, with large effects no significant difference between CAF and CTC. CTC also demonstrated significantly faster pro-target RT compared to PLA (−4.8%, *p* = 0.03, d = −0.47), revealing a moderate effect, while no significant differences were found between PLA and CAF or between CAF and CTC. Both CAF and CTC conditions demonstrated significantly lower pro-target CVRT (CAF: −14.1%, *p* = 0.03, d = −0.44 and CTC: −23.8%, *p* < 0.01, d = −0.84) and anti-target CVRT (CAF: −54.5%, *p* < 0.01, d = −0.41 and CTC: −43.9%, *p* < 0.01, d = −0.75), with moderate to large effects and no significant differences between CAF and CTC for either pro-target or anti-target CVRT. The CTC condition also had significantly faster anti-target RT compared to PLA with a moderate effect (−5.6%, *p* = 0.01, d = −0.56), while no significant differences were observed between PLA and CAF or between CAF and CTC. No significant main effects of time were observed for any measure (p’s > 0.05).

### 3.5. 2-back performance

Table 3 shows condition mean performance changes from baseline on Two-Back performance measures at each evaluation time point. No significant condition-by-time effects were observed. Main effects of condition were observed for total accuracy (*p* < 0.01), %Errors for target trials (*p* < 0.01), %Errors for non-target trials (*p* < 0.01), RT for non-target trials (*p* = 0.04), and CVRT to non-target objects (*p* < 0.01). Compared to the PLA condition, the CTC condition demonstrated significantly better accuracy on the Two-Back task (+7.3%, *p* < 0.01, d = 0.69), indicating a moderate effect, with no significant differences between CAF and PLA or between CAF and CTC. Both CAF and CTC conditions committed significantly fewer errors than PLA on target trials (CAF: −21.4%, *p* < 0.01, d = −0.79 and CTC: −17.5%, *p* < 0.01, d = −0.64), reflecting moderate to large effects, and non-target errors (CAF: −28.8%, *p* = 0.03, d = −0.44 and CTC: −43.3%, *p* < 0.01, d = −0.67), also indicating moderate effects, with no significant differences between CAF and CTC for target or non-target errors. Target reaction times were significantly faster in the CTC condition compared to PLA (−4.3%, *p* = 0.03, d = −0.24), a small effect, with no significant differences between CAF and PLA or between CAF and CTC. The CTC condition also showed significantly lower non-target CVRT compared to PLA (−12.0%, *p* < 0.01, d = −0.86) and CAF (−9.8%, *p* < 0.01, d = −0.63), suggesting moderate to large effects, with no significant difference between CAF and PLA. Main effects of time were observed for reaction times to both target (*p* < 0.01) and non-target objects (*p* < 0.01). Post-hoc analysis revealed significantly faster target and non-target reaction times compared to post-supplement evaluation at immediate post-exercise (target: −6.6%, *p* = 0.02, d = −0.81; non-target: −7.9%, *p* < 0.01, d = −0.77), indicating moderate to large effects across both target types, with no significant difference between the immediate and 30-min post-exercise time points. No other main effects for time were observed (ps > 0.05).

**Table 3.** Descriptive characteristics at each timepoint for the two-back task total accuracy, target errors (%), reaction time (RT, ms) for targets, coefficient of variation for reaction time of targets, non-target errors (%), RT for non-targets (ms), coefficient of variation for RT for non-targets. Percent changes were calculated as an average of individual percentage changes at each time point relative to baseline values. For condition effects, A = CAF, B = CTC, and C = PLA. For time effects, numbers represent time points in sequential order.

	CAF					CTC					PLA					Condition		Time		Interaction	
	Mean	SD	*d*	%Δ Mean	%Δ SD	Mean	SD	*d*	%Δ Mean	%Δ SD	Mean	SD	*d*	%Δ Mean	%Δ SD	p	η^2^	p	η^2^	p	η^2^
**Total Accuracy**																** < 0.001**	**0.087**	0.476	0.001	0.651	0.014
1. Baseline	76.4	16.0	-	-	-	77.2	13.1	-	-	-	80.6	12.1	-	-	-	A = B & A > C					
2. Post-Supp	79.2	11.8	-	0.7	8.5	80.5	11.5	-	4.0	8.0	78.1	14.2	-	−2.0	6.7	B = A & B > C					
3. Post-Ex	83.2	11.5	−0.88	4.5	8.5	81.5	11.7	−0.01	4.6	16.0	77.4	17.9	−0.36	−1.3	9.6						
4. 30-m Post-Ex	82.8	10.6	−0.39	1.8	6.4	79.8	12.9	−0.21	6.1	10.0	76.2	15.1	−0.01	−3.8	11.5						
**Error % (Targ)**																** < 0.001**	**0.112**	0.819	0.002	0.788	0.010
1. Baseline	46.3	18.8	-	-	-	47.9	20.4	-	-	-	40.1	16.0	-	-	-	A = B & A < C					
2. Post-Supp	38.3	22.7	-	−18.8	27.4	39.8	18.7	-	−23.7	25.8	41.5	17.8	-	−2.6	24.8	B = A & B < C					
3. Post-Ex	38.9	22.7	0.25	−25.0	27.4	38.2	17.8	−0.14	−18.9	117.9	42.1	23.5	−0.11	−2.2	33.1						
4. 30-m Post-Ex	35.7	20.3	0.27	−24.3	20.1	42.0	22.1	−0.35	−14.0	32.4	45.7	19.9	−0.31	0.8	29.3						
**RT (Targ)**																0.185	0.012	** < 0.001**	**0.108**	0.576	0.017
1. Baseline	599.2	165.9	-	-	-	557.2	106.9	-	-	-	563.2	159.0	-	-	-						
2. Post-Supp	573.4	146.5	-	−4.6	13.9	543.2	162.3	-	−1.9	20.2	538.2	180.7	-	−3.6	13.6			** > 3 & > 4**			
3. Post-Ex	497.4	158.1	0.83	−15.1	8.4	481.1	126.4	0.80	−17.5	9.5	508.1	144.5	0.56	−10.0	12.2			** < 2 & = 4**			
4. 30-m Post-Ex	517.2	177.3	0.63	−12.7	11.9	506.4	147.6	0.44	−10.9	9.6	516.2	159.1	0.28	−6.3	15.2			** < 2 & = 3**			
**CVRT (Targ)**																0.058	0.033	0.677	0.005	0.481	0.02
1. Baseline	31.1	14.0	-	-	-	24.7	8.9	-	-	-	27.8	10.1	-	-	-						
2. Post-Supp	25.6	9.3	-	−6.2	24.6	23.2	9.8	-	−8.4	39.5	23.6	8.9	-	−15.5	20.3						
3. Post-Ex	26.5	11.1	0.21	−13.4	29.3	25.7	10.1	−0.15	2.5	45.8	24.6	10.4	−0.30	−7.7	38.2						
4. 30-m Post-Ex	25.5	10.1	0.23	−12.9	39.5	28.8	8.4	−0.54	11.1	37.3	24.2	10.4	−0.30	−12.2	26.0						
**Error % (NonTarg)**																** < 0.001**	**0.083**	0.485	0.008	0.694	0.013
1. Baseline	18.6	17.2	-	-	-	15.3	11.4	-	-	-	14.5	13.1	-	-	-	A = B & A < C					
2. Post-Supp	17.0	11.7	-	11.8	49.2	14.3	11.8	-	−9.2	52.3	17.0	15.6	-	24.9	72.1	B = A & B < C					
3. Post-Ex	12.0	11.4	0.86	−18.0	51.2	14.1	12.4	0.15	−15.9	56.9	18.6	19.2	−0.06	31.8	82.0						
4. 30-m Post-Ex	12.6	12.0	0.36	−3.5	54.6	15.4	13.6	0.33	−27.9	43.1	19.1	17.1	0.04	20.1	77.2						
**RT (NonTarg)**																**0.037**	**0.038**	** < 0.001**	**0.136**	0.815	0.010
1. Baseline	590.9	152.0	-	-	-	545.9	139.4	-	-	-	564.3	161.3	-	-	-	A = B & A = C		** > 3 & > 4**			
2. Post-Supp	553.8	148.5	-	−7.1	7.5	482.0	146.7	-	−9.3	5.7	536.5	163.6	-	−5.2	11.9	B = A & B < C		** < 2 & = 4**			
3. Post-Ex	520.7	142.9	0.63	−12.2	8.2	473.8	133.6	0.98	−17.3	9.3	484.2	132.6	0.97	−12.6	8.9			** < 2 & = 3**			
4. 30-m Post-Ex	498.6	146.6	1.05	−16.1	7.6	462.8	147.9	0.96	−16.5	8.0	475.7	148.3	0.79	−12.7	12.4						
**CVRT (NonTarg)**																** < 0.001**	**0.103**	0.790	0.003	0.441	0.022
1. Baseline	29.7	11.9	-	-	-	30.9	9.7	-	-	-	29.7	9.3	-	-	-	A > B& A = C					
2. Post-Supp	28.7	6.9	-	1.7	14.7	27.2	7.7	-	−10.9	7.7	27.8	7.0	-	−1.0	12.8	B < A & B < C					
3. Post-Ex	27.5	7.3	−0.05	2.3	21.3	27.8	7.4	0.01	−10.0	13.6	30.0	9.4	−0.14	1.9	11.1						
4. 30-m Post-Ex	26.0	7.2	0.40	−5.6	18.9	28.3	8.9	−0.05	−10.1	13.6	29.3	7.6	−0.21	4.1	22.1						

### 3.6. Heart rate variability

Table 4 shows condition performance changes from baseline on HRV measures of cardio-autonomic function at each time point. No significant condition-by-time effects were observed. No significant main effect of condition was observed for any HRV measure. Main effects of time for average HR (*p* < 0.01), average NN interval (*p* < 0.01), SDNN (*p* < 0.01), RMSSD (*p* < 0.01), and CVNN (*p* < 0.01) were observed. All three conditions demonstrated significantly lower HR during the post-supplement cognitive evaluation compared to the baseline cognitive evaluation (−10.5%, *p* < 0.01, d = −1.27), indicating large effects. However, for all three conditions HR was significantly higher both immediately post-exercise (+33.6%, *p* < 0.01, d = 3.02), and 30 min post-exercise (+15.2%, *p* < 0.01, d = 1.50), representing large effects. Compared to post-supplementation, all three conditions had significantly higher HR immediately post-exercise (+44.1%, *p* < 0.01, d = 3.94) and 30 min post-exercise (+25.7%, *p* < 0.01, d = 2.52), both suggesting large effects. Finally, HR significantly decreased for all three conditions at 30 min post-exercise compared to immediately post-exercise (−18.4%, *p* < 0.01, d = −1.46), with a large effect.

**Table 4.** Descriptive characteristics at each timepoint for heart rate (HR, bpm), NN-interval, SDNN, RMSSD, and CVNN. Percent changes were calculated as an average of individual percentage changes at each time point relative to baseline values. For time effects, numbers represent time points in sequential order.

	CAF					CTC					PLA					Condition		Time		Interaction	
	Mean	SD	*d*	%Δ Mean	%Δ SD	Mean	SD	*d*	%Δ Mean	%Δ SD	Mean	SD	*d*	%Δ Mean	%Δ SD	p	η^2^	p	η^2^	p	η^2^
**HR**																0.893	0.001	** < 0.001**	**0.715**	0.649	0.018
1. Resting	69.9	10.3	-	-	-	71.2	13.4	-	-	-	71.3	11.4	-	-	-						
2. Baseline	67.9	9.0	-	−3.8	9.9	68.2	11.7	-	−3.6	8.2	69.3	10.9	-	−3.8	6.5			** > 3, < 4 & < 5**			
3. Post-Supp	60.0	7.1	1.45	−15.5	6.9	62.1	10.7	1.66	−15.3	9.6	63.6	8.4	0.98	−12.0	8.4			** < 2. < 4 & < 5**			
4. Post-Ex	93.9	8.2	−3.51	31.8	10.6	93.4	11.3	−2.97	31.0	16.1	95.5	11.5	−2.73	26.6	13.1			** > 2, > 3 & > 5**			
5. 30-m Post-Ex	80.5	8.8	−2.02	12.4	8.5	79.6	10.7	−1.93	10.8	14.1	83.1	12.8	−1.30	11.0	12.6			** > 2, > 3, & < 4**			
**NN**																0.215	0.013	** < 0.001**	**0.777**	0.152	0.040
1. Resting	889.2	145.5	-	-	-	890.9	181.6	-	-	-	876.0	154.0	-	-	-						
2. Baseline	909.6	125.7	-	2.0	11.4	915.7	155.9	-	4.2	7.6	897.0	139.6	-	3.9	6.9			** < 3, > 4 & > 5**			
3. Post-Supp	1028.1	125.3	−1.64	20.9	9.3	1014.5	169.9	−1.86	16.0	10.7	974.1	131.3	−0.94	11.9	8.0			** > 2, > 4 & > 5**			
4. Post-Ex	651.6	59.4	2.40	−24.9	8.1	658.6	82.1	3.81	−25.8	9.0	641.7	79.1	-	−24.9	8.4			** < 2, < 3 & < 5**			
5. 30-m Post-Ex	762.0	83.8	1.23	−10.6	6.2	775.2	104.4	3.05	−11.6	7.1	743.71	112.83	2.04	−13.2	8.9			** < 2, < 3 & > 4**			
**SDNN**																0.510	0.006	** < 0.001**	**0.473**	0.886	0.01
1. Resting	95.1	39.7	-	-	-	101.4	60.5	-	-	-	92.7	41.4	-	-	-						
2. Baseline	83.6	27.8	-	−8.9	32.7	86.7	35.8	-	−6.9	26.9	86.0	30.3	-	−5.5	25.8			** > 3, > 4 & > 5**			
3. Post-Supp	107.8	25.4	−0.63	16.4	37.6	116.3	45.5	−0.67	20.5	44.4	103.9	35.4	−0.59	17.3	29.8			** > 2, > 4 & > 5**			
4. Post-Ex	46.0	10.7	1.15	−47.9	20.4	50.1	18.1	2.01	−49.6	15.8	41.5	16.0	1.70	−52.9	16.3			** < 2, < 3 & < 5**			
5. 30-m Post-Ex	64.0	23.3	0.64	−31.4	20.0	71.2	23.4	0.49	−21.5	25.6	57.8	27.6	1.12	−36.2	19.3			** < 2, < 3 & > 4**			
**RMSSD**																0.195	0.014	** < 0.001**	**0.599**	0.971	0.006
1. Resting	66.9	40.5	-	-	-	75.0	54.8	-	-	-	70.3	45.2	-	-	-						
2. Baseline	62.5	34.3	-	3.8	31.2	70.0	38.3	-	6.7	37.4	64.2	34.1	-	1.8	24.1			** < 3, > 4 & > 5**			
3. Post-Supp	83.6	31.5	−1.62	46.4	38.0	90.1	31.5	−0.59	41.3	60.4	83.3	43.3	−1.15	39.6	50.5			** > 2, > 4 & > 5**			
4. Post-Ex	23.2	8.1	3.09	−57.1	18.1	27.6	15.2	2.07	−60.2	18.4	19.1	10.8	4.15	−68.9	10.7			** < 2, < 3 & < 5**			
5. 30-m Post-Ex	44.6	27.7	1.36	−26.3	18.1	51.5	24.8	1.04	−26.6	28.2	38.6	27.7	2.09	−40.7	20.0			** < 2, < 3 & > 4**			
**CVNN**																0.112	0.020	** < 0.001**	**0.175**	0.815	0.013
1. Resting	10.5	3.6	-	-	-	11.0	5.7	-	-	-	10.4	4.1	-	-	-						
2. Baseline	9.2	2.5	-	−11.1	27.0	9.3	2.9	-	−7.2	25.6	9.5	2.5	-	−3.4	31.2			** = 3, > 4 & = 5**			
3. Post-Supp	10.4	2.2	−0.27	−1.1	24.6	11.6	4.8	−0.37	11.0	46.6	10.5	3.0	−0.21	1.0	20.7			** = 2, > 4 & > 5**			
4. Post-Ex	7.1	1.7	0.61	−29.8	24.6	7.5	2.2	0.69	−24.7	27.0	6.4	1.9	0.96	−36.0	20.5			** < 2, < 3 & = 5**			
5. 30-m Post-Ex	8.3	2.6	0.26	−20.5	19.4	9.2	2.8	0.07	−8.6	29.5	7.6	2.8	0.47	−21.7	25.8			** = 2, < 3 & = 4**			

Compared to the baseline cognitive evaluation, all participants had significantly greater average NN intervals (+12.9%, *p* < 0.01, d = 1.38), and SDNN (+25.1%, *p* < 0.01, d = 0.76), indicating a moderate to large effect during the post-supplement cognitive evaluation. However, for all participants both NN intervals and SDNN were significantly smaller immediately post-exercise (NN: −28.6%, d = −3.34; SDNN: −43.1%, d = −1.84) and 30 min post-exercise (NN: −15.2%, d = −1.87; SDNN: −22.63%, d = 0.89), revealing large effects. Compared to post-supplement levels, all participants had significantly smaller NN intervals (−41.5%, *p* < 0.01, d = −4.51) and SDNN (−68.2%, *p* < 0.01, d = −2.35) immediately post-exercise, as well as significantly smaller NN intervals (−28.1%, *p* < 0.01, d = −3.20) and SDNN (−47.5%, *p* < 0.01, d = −1.56) 30 min post-exercise, all of which represented large effects. NN intervals (13.4%, *p* < 0.01, d = 1.70) and SDNN (20.4%, *p* < 0.01, d = 1.02), were significantly greater for all participants at 30 min post-exercise compared to immediately post-exercise, both with large effects.

RMSSD significantly increased for all three conditions after supplementation compared to baseline cognitive evaluation (38.3%, *p* < 0.01, d = 0.93), but significantly decreased immediately post-exercise (−66.1%, *p* < 0.01, d = −2.67) and at 30 min post-exercise (−35.3%, *p* < 0.01, d = −1.29), indicating large effects. RMSSD was also significantly lower for all three conditions immediately post-exercise (−104.45%, *p* < 0.01, d = −2.82) and 30 min post-exercise (−73.6%, *p* < 0.01, d = −1.90) when compared to post-supplementation, though it increased between the immediate and 30-min post-exercise time points (+30.9%, *p* < 0.01, d = 1.53), representing large effects.

For all three conditions CVNN was significantly decreased immediately post-exercise compared to baseline (−23.0%, *p* < 0.01, d = −0.88), but no significant difference between baseline and either post-supplement or 30 min post-exercise. Compared to post-supplementation, CVNN was significantly lower for all three conditions immediately post-exercise (−33.8%, *p* < 0.01, d = −1.18) and 30 min post-exercise (−20.5%, *p* < 0.01, d = −0.70), all with moderate to large effects but with no significant difference between the two post-exercise time points.

### 3.7. Lactate

Table 5 shows condition blood lactate concentration changes from baseline throughout each experimental session. No significant condition-by-time effects were observed. No main effect of condition was observed. However, there was a main effect of time for blood lactate concentration (*p* < 0.01). Post-hoc analysis revealed that compared to immediately post-exercise, all three conditions had significantly lower blood lactate levels at both 5 min post-exercise (−158.0%, *p* < 0.01, d = −0.80) and 10 min post-exercise (−294.5%, *p* < 0.01, d = −1.67), indicating large effects. Additionally, blood lactate levels for all three conditions were significantly lower at the 10-min post-exercise time point compared to the 5-min post-exercise sample (−136.5%, *p* < 0.01, d = −0.92), revealing a large effect.

**Table 5.** Descriptive characteristics at each timepoint for blood lactate concentrations (mM). Percent changes were calculated as an average of individual percentage changes at each time point relative to baseline values. For time effects, numbers represent time points in sequential order.

	CAF					CTC					PLA					Condition		Time		Interaction	
	Mean	SD	*d*	%Δ Mean	%Δ SD	Mean	SD	*d*	%Δ Mean	%Δ SD	Mean	SD	*d*	%Δ Mean	%Δ SD	p	η^2^	p	η^2^	p	η^2^
**Blood Lactate (mM)**																0.066	0.031	** < 0.001**	**0.331**	0.994	0.001
1. Baseline	0.8	0.3	-	-	-	0.9	0.3	-	-	-	0.9	0.3	-	-	-						
2. Post-Supp	5.0	1.4	-	500.6	234.0	5.0	1.6	-	501.5	199.2	4.6	1.5	-	422.4	224.7			** > 3 & > 4**			
3. Post-Ex	3.6	1.2	0.66	331.9	181.7	3.5	1.3	0.77	346.7	175.4	3.2	1.3	0.63	271.9	166.6			** < 2 & > 3**			
4. 30-m Post-Ex	2.4	0.8	1.38	191.6	123.2	2.2	0.7	1.67	201.5	115.0	2.3	1.0	1.31	147.9	113.2			** < 2 & < 3**			

## 4. Discussion

The present findings indicate that both 300 mg of caffeine and a combination of 150 mg of caffeine with 150 mg of theacrine (CTC) significantly enhance cognitive performance, both before and after physically fatiguing exercise in a tactical population. This is consistent with previous findings in high-level soccer players demonstrating the ability of caffeine and CTC to improve cognitive functioning following fatiguing exercise [7]. Notably, the present study extends these findings in that CTC not only improved general cognitive performance but also reduced response variability. CVRT is believed to be an indicator of nervous system functional integrity, and higher CVRT may reflect inefficient control of arousal, and cognitive-energetics, resulting in lapses in sustained and top-down cognitive control [17,18]. In the context of the present study, the improvements in CVRT following high-intensity exercise within the CTC condition would suggest that a combination of theacrine and caffeine does indeed mitigate deficits in attention and inhibitory control.

Furthermore, in addition to working memory, the 2-back task requires sustained attention and cognitive inhibition to produce accurate responses. Thus, when considered with the reduced CVRTs observed for the CTC only, our findings suggest that CTC has significant benefits beyond high-dose caffeine in multiple aspects of cognitive-behavioral performance critical to tactical success.

Additionally, we observed that both caffeine and CTC supplementation improves accuracy across several cognitive tasks. Specifically, these cognitive performance improvements appear driven by more efficient attentional resource allocation (i.e. inhibition to anti-saccade targets, avoidance of distractor objects, and recognition of target objects). Previous research suggests that higher order executive functions are the most susceptible to influence by mental and physical exertion [19,20–22]. In this context, the present findings suggest that supplementation with CAF and CTC may serve to mitigate losses in the ability to accurately identify targets and avoid non-targets following cognitive and physical fatigue.

Interestingly, we did not observe any significant differences in any measure of HR and HRV between the conditions. As a psychoactive compound that can facilitate catecholamine release, caffeine typically elicits increases in heart rate and blood pressure [20]. However, in habitual caffeine consumers, alterations in HR, hemodynamics, and HRV are not as heavily affected following consumption [21]. As the participants were required to be habitual caffeine consumers, it is likely the participants were desensitized to the heightened cardiovascular side effects of caffeine. Future research in non-habituated caffeine users may be necessary to further elucidate the cardiovascular and hemodynamic benefits to CTC supplementation as compared to caffeine.

The present results are particularly relevant for tactical populations engaged in prolonged tasks that are accompanied by high degrees of physiological, psychological, and environmental stress. In such scenarios, personnel must maintain high levels of cognitive function and response consistency despite physical fatigue from stressors such as carrying heavy equipment, covering large distances on foot, and enduring harsh environmental conditions. Our results suggest that CTC supplementation could be strategically implemented before the commencement of such operations to prevent a decline in aspects of cognition that are critical for rapid decision-making, effective communication, and overall operational success. While both caffeine and CTC supplementation improved cognitive performance in a rested state, it was particularly notable that the improvements observed with CTC were sustained following fatiguing high-intensity exercise. This divergent effect underscores the potential of CTC as an effective ergogenic aid for cognitive resilience in high-stress environments requiring high levels of physical exertion. Moreover, these findings may extend beyond the military context in which cognitive and physical endurance are simultaneously demanded, such as in long-duration team sports and emergency response situations. [23,24]

The present study does contain limitations which must be considered. For example, military age participants and the specific physical and cognitive tasks employed should be considered when generalizing these findings. Although participants completed a single familiarization session to reduce learning effects, residual practice effects across sessions cannot be completely ruled out. However, experimental conditions were randomized to minimize any systematic bias in task performance due to session order. Additionally, although participants were required to abstain from caffeine for 24 hours prior to each visit, we did not stratify individuals based on habitual caffeine intake or assess individual differences in caffeine sensitivity. These factors may have influenced response variability, and future research may benefit from incorporating habitual use stratification. Recent research, however, has indicated that the effects of caffeine are robust regardless of habitual intake [3]. Although no significant differences in HRV were observed between conditions, it is possible that the study was underpowered to detect small-to-moderate treatment effects in this outcome. Additionally, HRV is highly sensitive to individual differences in autonomic tone, time of day, and recovery state. While sessions were scheduled consistently for each participant, minor variability in these factors may have influenced HRV outcomes. As such, null findings should be interpreted with caution, and future studies may benefit from larger sample sizes or more controlled autonomic testing environments. Additionally, the CV was high for a small number of outcome measures, which suggests that results for these specific measures should be interpreted with caution. Eye tracking in particular can be influenced by several factors such as changes in corneal reflection, body movement induced ocular drift, and excessive blinking [24]. However, the majority of outcome measures across all tasks showed CV values within accepted thresholds for reliability ( <30%), indicating overall consistency and stability.

The present study also contained several strengths. Notably, we utilized a double-blind, placebo-controlled, randomized within-subjects design with sessions separated by ≥96 hours. In addition, each experimental session was of a duration sufficient to allow for peak plasma concentrations of theacrine to be achieved [25]. We also included a comprehensive battery of ecologically valid cognitive-behavioral measures. Lastly, this study represents the first to study the effects of CTC supplementation in a tactical population typically ingesting high levels of caffeine [23].

Future studies should consider exploring the effects of CTC in a variety of populations with different external stressors, such as heat stress or more prolonged and fatiguing bouts of exercise to determine the robustness and applicability of our results across different settings. Future research should also examine the optimal dosing and timing strategies in addition to the potential long-term effects of regular CTC use. Additionally, investigating the underlying biochemical pathways involved in the observed cognitive enhancements could provide deeper insight into how these compounds interact at a neurohumoral level, and inform more targeted intervention strategies.

## 5. Conclusion

Our study highlights the efficacy of caffeine alone and in combination with theacrine for enhancing cognitive performance, both before and after fatiguing exercise. Importantly, our results highlight certain benefits of lower-dose caffeine combined with theacrine beyond higher-dose caffeine for critical functions such as attentional control, response consistency, and working memory. Thus, our findings support the potential for the combined ingestion of caffeine and theacrine as a superior cognitive enhancer under physical stress, offering valuable insights for enhancing cognitive resilience in high-stress professions.
